# The multifaceted role of insulin-like growth factor binding protein 7

**DOI:** 10.3389/fcell.2024.1420862

**Published:** 2024-07-16

**Authors:** Li Chen, Linhu Hui, Jun Li

**Affiliations:** Department of Immunology, Center of Immunomolecular Engineering, Innovation and Practice Base for Graduate Students Education, Zunyi Medical University, Zunyi, China

**Keywords:** acute kidney injury, IGFBP7, insulin-like growth factor, reproduction, tumor

## Abstract

Insulin-like growth factor binding protein 7 (IGFBP7) serves as a crucial extracellular matrix protein, exerting pivotal roles in both physiological and pathological processes. This comprehensive review meticulously delineates the structural attributes of IGFBP7, juxtaposing them with other members within the IGFBP families, and delves into the expression patterns across various tissues. Furthermore, the review thoroughly examines the multifaceted functions of IGFBP7, encompassing its regulatory effects on cell proliferation, apoptosis, and migration, elucidating the underlying mechanistic pathways. Moreover, it underscores the compelling roles in tumor progression, acute kidney injury, and reproductive processes. By rigorously elucidating the diverse functionalities and regulatory networks of IGFBP7 across various physiological and pathological contexts, this review aims to furnish a robust theoretical framework and delineate future research trajectories for leveraging IGFBP7 in disease diagnosis, therapeutic interventions, and pharmaceutical innovations.

## 1 Introduction

Insulin-like growth factor binding protein 7 (IGFBP7), as a crucial member of the IGFBP family, has been extensively investigated and recognized for its significant roles in cellular biology and pathophysiology. Serving as an extracellular matrix protein, IGFBP7 not only participates in regulating fundamental biological processes such as cell proliferation (Xia et al., 2020), apoptosis (Tang et al., 2021), and migration (Hong et al., 2023) but also exerts important regulatory effects in tumor development (Yi et al., 2022; Artico et al., 2023), angiogenesis (Bracun et al., 2022; Lam et al., 2022; Liu et al., 2023; Tan et al., 2023), renal diseases (Waskowski et al., 2021; Chapman et al., 2023; Hu et al., 2023; Stanski et al., 2023), and reproduction (Wandji et al., 2000; Huang et al., 2021; Wu et al., 2022). In recent years, continuous research focus on the relationship between the structure and function of IGFBP7, as well as its mechanistic involvement in various diseases have gradually been acknowledged. However, numerous mysteries persist regarding the functional mechanisms of IGFBP7, its potential applications in disease diagnosis, treatment, and drug development. Therefore, this review aims to systematically summarize the recent advances in the study of IGFBP7, encompassing its structural characteristics, expression pattern, as well as its functional and mechanistic roles in different physiological and pathological processes. The comprehensive understanding of IGFBP7’s biological functions provided herein is intended to lay a theoretical foundation and guide future research directions for its further development in clinical applications.

## 2 Structure and function of the IGFBP family

IGFBPs, a family of proteins that bind to insulin-like growth factors (IGFs) and regulate their biological activity, play a crucial role in the IGF signaling pathway (Ma et al., 2023). By binding to IGFs, IGFBPs modulate their biological activity and availability, prolonging their half-life *in vivo* and regulating their access to IGF receptors, thus impacting the activity of the IGF signaling pathway and regulating biological processes such as cell growth, proliferation, and apoptosis (Baxter, 2023; Galal et al., 2023; Werner, 2023). Based on their different affinities for IGF, IGFBPs are divided into two classes: high-affinity binding proteins (IGFBP1-6) and low-affinity binding proteins (IGFBP-rP1-10).

IGFBPs are a family of proteins characterized by multiple conserved domains. They typically consist of three distinct domains: the N-terminal domain, the C-terminal domain, and the central domain. The N-terminal domain contains approximately 16–18 conserved cysteine residues, including a common IGFBP motif (GCGCCXXC), which is a key region for binding to IGFs (Vorwerk et al., 2002). In contrast, the C-terminal domain usually contains about six conserved cysteine residues, with potential variations among different members of the IGFBP family (Zhou et al., 2023). The central domain, also known as the binding domain, exhibits structural differences from other domains and typically contains glycosylation and phosphorylation sites, which can influence the activity and stability of IGFBPs. The central domain mediates the binding of IGFBPs to IGFs, thereby regulating the biological activity of IGFs and the activation of cellular signaling pathways (Fowlkes et al., 1997).

The IGFBP-rPs, including IGFBP-rP1 to IGFBP-rP10, share structural and functional similarities with IGFBPs. IGFBP-rP1, initially named *IGFBP7*, was the first discovered IGFBP-related protein component due to its ability to connect with IGF via the N-terminal domain (Song et al., 2021). *IGFBP7* has been cloned from various cellular systems and is known by multiple names such as *mac25* (Kato, 2000), tumor adhesion factor (Albelda, 1993), prostate stromal factor (Yarosh et al., 2015), and angiostatin (Jin et al., 2020). Structurally, *IGFBP7* differs significantly from other IGFBPs, particularly in its C-terminal domain, which lacks conserved cysteine residues, possessing only one cysteine residue (Oh et al., 1996). Moreover, IGFBP7 exhibits 100-fold lower affinity for binding to IGF-1 and is the only member of the family that binds insulin with strong affinity, limiting its binding to insulin receptors (Yamanaka et al., 1997). Unlike IGFBP3 and IGFBP5, IGFBP7 is not subject to glycosylation or phosphorylation effects and is distinguished from other IGFBPs by its regulation mechanisms at the RNA and DNA levels (Kutsukake et al., 2008). These structural and post-translational modification differences suggest that IGFBP7 may possess unique functions independent of IGF.

## 3 Expression of *IGFBP7*

**FIGURE 1:**
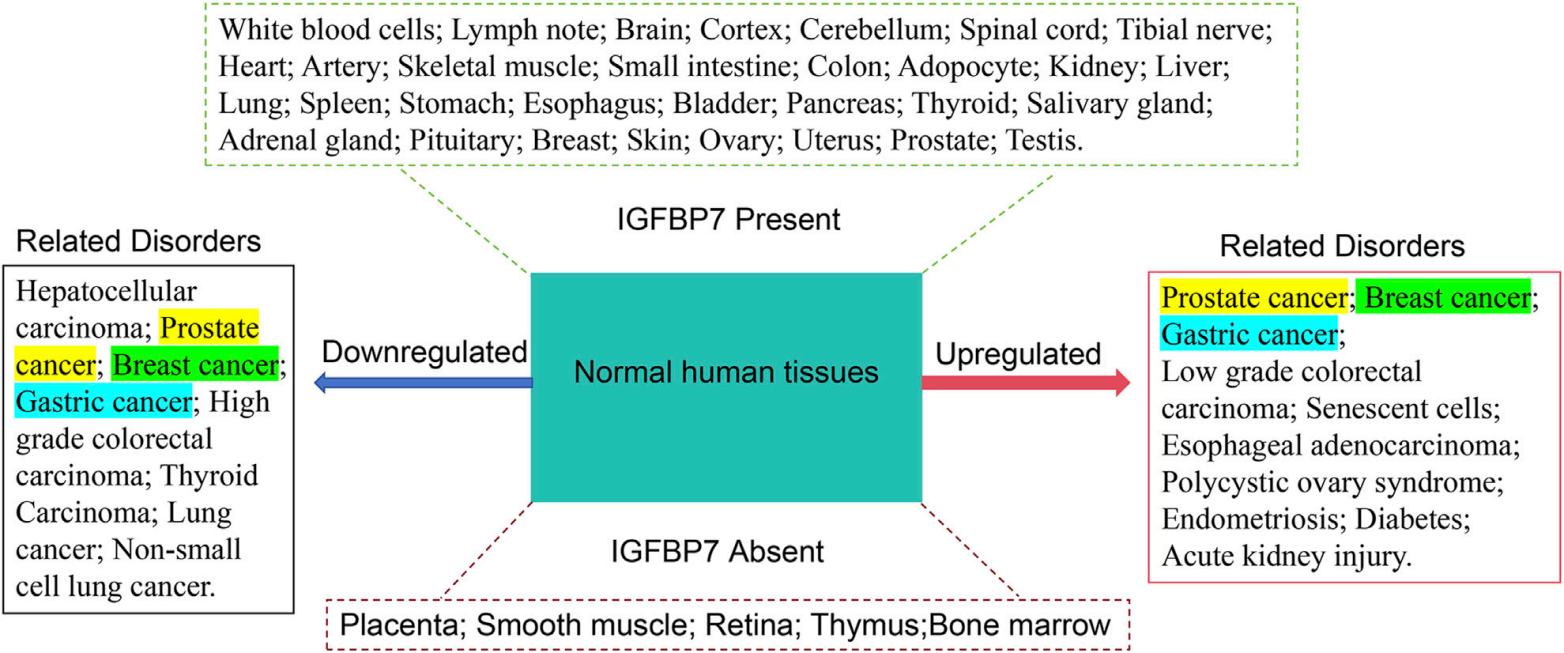
Expression of IGFBP7 in various tissues and its association with diseases related to its downregulation and upregulation. The left side lists diseases associated with downregulation of IGFBP7 expression, while the right side lists diseases associated with upregulation of IGFBP7 expression. The upper part shows tissues and organs where IGFBP7 expression is detected, and the lower part shows tissues and organs where IGFBP7 expression is not detected. Diseases marked with the same color indicate the same disease. Data are sourced from transcriptome sequencing data in the GEO and GeneCards databases.

*IGFBP7* expression was detected in various normal tissues (Figure 1) including brain, liver, heart, small intestine, spleen, kidney, placenta, lung, skeletal muscle, thymus, prostate, testis, ovary, pancreas, and colon (Hwa et al., 1998). Immunohistochemical analysis revealed strong positive staining of IGFBP7 in peripheral nerves, respiratory cilia, epididymis, and fallopian tubes; smooth muscle cells in intestines, bladder, prostate, and endothelial cell walls also exhibited strong positive staining (Degeorges et al., 2000). Conversely, lymphocytes, plasma cells, and adipocytes displayed negative staining (Artico et al., 2021). Within the kidneys, stronger staining was observed in the epithelium of distal tubules compared to proximal tubules (Sekiuchi et al., 2012). Moreover, cells from the reticular zone and glomerular zone showed stronger staining than those from the cortical zone, with some studies indicating stronger expression of IGFBP7 in proximal tubules and localization along the brush border of certain proximal convoluted tubules (Emlet et al., 2017). In the liver, analysis via serial analysis of gene expression revealed that activated stellate cells were the major contributors to *IGFBP7* expression (Degeorges et al., 2000). Notably, compared to isolated activated stellate cells, *IGFBP7* exhibited lower expression throughout the entire liver. Immunohistochemical studies conducted on human prostate tissue (normal) demonstrated universally intense staining (Degeorges et al., 1999). IGFBP7 is also detectable in various body fluids such as serum, urine, cerebrospinal fluid, and amniotic fluid of pregnant women (Anderlová et al., 2022). The cell-specific differential expression pattern of *IGFBP7* within tissues may suggest its potential specific functions in these organs.

## 4 Functional mechanisms of IGFBP7

IGFBP7, a novel member of the IGFBP superfamily, possesses a unique molecular structure characterized by a conserved N-terminal domain similar to other IGFBPs, as well as distinctive Kazal-type serine protease inhibitor domains and immunoglobulin-like C2 domains (Yamanaka et al., 1997). Apart from its canonical role in modulating the effects of IGFs, IGFBP7 independently regulates cellular processes such as apoptosis, proliferation, and migration (Kim et al., 1997). In particular, IGFBP7 is implicated in cell adhesion and tumor cell proliferation processes, with its N-terminal fragments post-degradation retaining cell membrane adhesion properties (Oh et al., 1996; Vorwerk et al., 2002). Studies have demonstrated an upregulation of *IGFBP7* expression in cells treated with TGF-β1 and retinoic acid (Oh, 1998). Additionally, IGFBP7 has been shown to bind to cell surface heparan sulfate, although this interaction may be influenced by the cleavage of IGFBP7 by pancreatic trypsin-like integral membrane serine protease, matriptase (Godfried Sie et al., 2012). Cleavage by matriptase at the P1 site, involving Arg or Lys residues, has been associated with breast cancer invasion and metastasis. Proteolytic cleavage, particularly at the N-terminus, including the heparin-binding domain, reduces heparin binding and IGF-1R occupancy (Werner, 2023).

Furthermore, researchers have observed co-localization of IGFBP7 with the basement membrane in the vasculature, and subsequent direct measurement of IGFBP7 binding to extracellular matrix proteins, revealing its ability to bind to Type IV collagen (Pen et al., 2007). Moreover, IGFBP7 was found to stimulate adhesion of human umbilical vein endothelial cells to Type IV collagen matrices, inducing morphological changes. St Croix et al. (2000) also identified a role for IGFBP7 in binding to Type IV collagen protein. They demonstrated elevated expression of *IGFBP7* compared to healthy endothelial cells, suggesting IGFBP7 as a potential tumor endothelial cell marker, as determined by serial analysis of gene expression.

## 5 The role of IGFBP7 in tumor development

The role of IGFBP7 in cancer has been a highly researched area of interest. Numerous studies have confirmed the association between *IGFBP7* and various cancers (Jin et al., 2020; Li et al., 2023), including hepatocellular carcinoma, breast cancer (Godina et al., 2021; Wilcox et al., 2021), esophageal cancer (Li et al., 2022), colorectal cancer, and prostate cancer (Singh et al., 2020). However, the role of IGFBP7 appears to exhibit a complex pattern across different types of cancer. Utilizing detection techniques such as qRT-PCR, immunohistochemistry, Northern blot, and Western blot, studies have revealed that *IGFBP7* expression is generally downregulated in hepatocellular carcinoma, melanoma, and lung cancer, while showing an upregulation trend in esophageal cancer. In breast, gastric, prostate, colorectal, and glioma cancers, some studies have reported upregulation of *IGFBP7* expression, while others have reported downregulation, indicating a dual role of IGFBP7 in cancer cell proliferation, progression, and prognosis (Lin et al., 2019). Furthermore, research on IGFBP7 has shown its ability to alter cancer cell sensitivity to chemotherapy drugs, suggesting its potential beneficial value in anticancer therapy (Roška et al., 2020; Tang et al., 2021). However, despite a wealth of studies elucidating the significant role of IGFBP7 in tumor development, its specific mechanisms and roles in different types of cancer still require further investigation.

**FIGURE 2:**
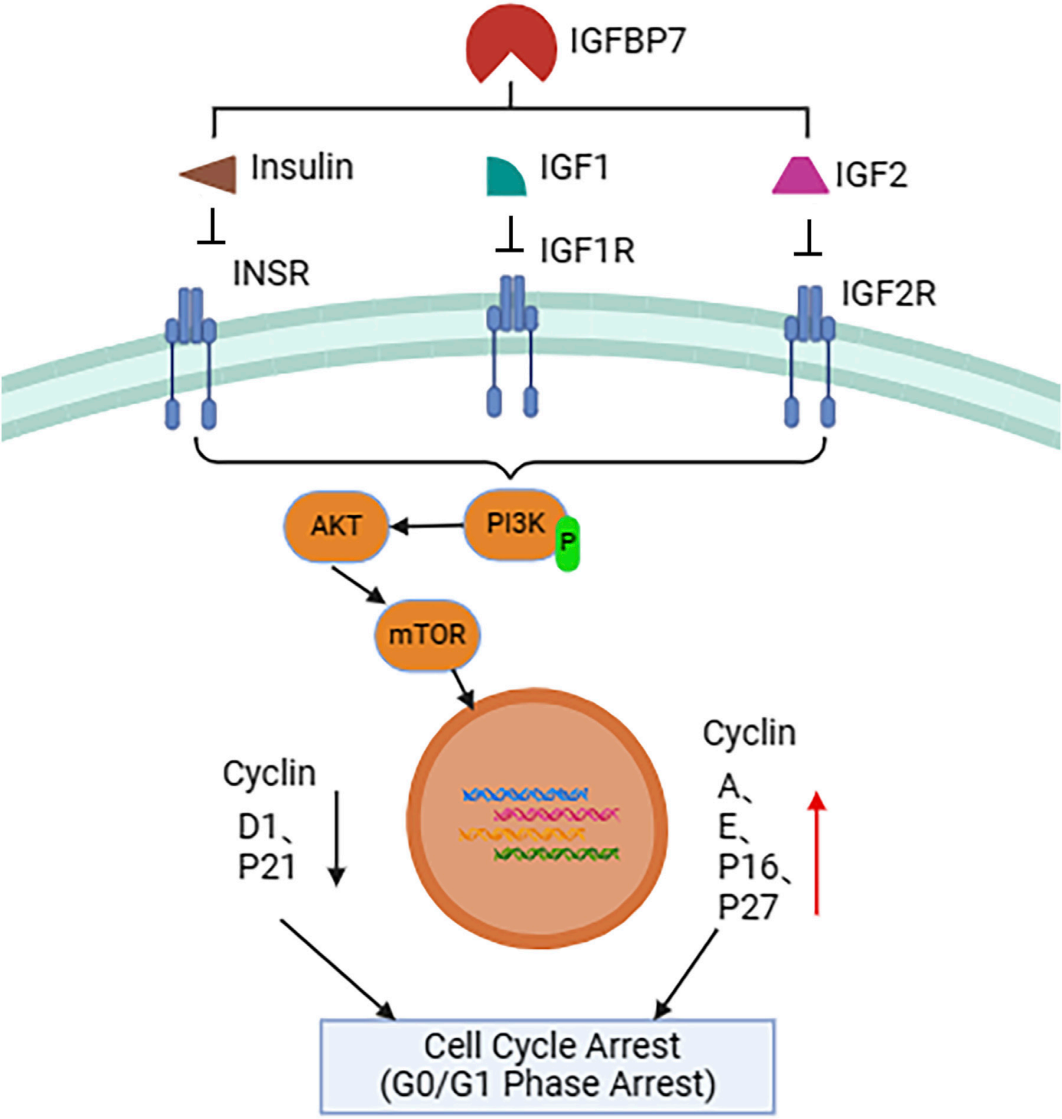
Schematic representation of IGFBP7-mediated regulation of the cell cycle. IGFBP7 interacts with insulin (INS), insulin-like growth factor 1 (IGF1), and insulin-like growth factor 2 (IGF2), inhibiting their binding to corresponding cell membrane receptors such as insulin receptor (INSR), IGF1 receptor (IGF1R), and IGF2 receptor (IGF2R). This process affects the activation of the intracellular PI3K-AKT-mTOR signaling pathway, ultimately leading to upregulation of nuclear cell cycle-related proteins A (Cyclin A), E (Cyclin E), P16 (CDKN2A), and P27 (CDKN1B), while downregulating D1 (Cyclin D1) and P21 (CDKN1A), resulting in cell cycle arrest at the G0/G1 phase. This IGFBP7-mediated cell cycle regulation mechanism may have significant implications for tumor growth and treatment.

IGFBP7 primarily exerts its anti-tumor effects by inhibiting tumor cell growth and accelerating tumor cell apoptosis (Figure 2). This is achieved through inhibition of the expression of cell cycle proteins D1 and p21, and promotion of the expression of cell cycle proteins A, E, p16, and p27, or by suppressing Akt kinase activity, leading to upregulation of cyclin-dependent kinase (CDK) inhibitory factors p27Kip1 and p21Cip1, thereby inducing cell cycle arrest at the G0/G1 phase. Overexpression of *IGFBP7* or addition of exogenous IGFBP7 in cell culture can induce cell cycle arrest at the G2 phase through non-IGF-1 receptor, AKT, and ERK pathways, subsequently leading to cell apoptosis (Sato et al., 2007; Wang et al., 2017; Zhang et al., 2019). Despite some conflicting conclusions, the majority of evidence currently suggests that IGFBP7 inhibits tumor cell growth and promotes tumor cell apoptosis, rendering it a potential candidate for tumor suppression. Overall, IGFBP7 exhibits a dual role in tumor development, inhibiting tumor cell growth and accelerating tumor cell apoptosis, thus emerging as a potential candidate for tumor suppression. However, further research is needed to elucidate its specific mechanisms and roles in different types of cancer.

## 6 The role of IGFBP7 in acute kidney injury

IGFBP7 has been proposed as a biomarker for acute kidney injury (AKI), aiming to enhance early detection, discrimination, and prognosis assessment, complementing serum creatinine and urine output (Meena et al., 2023; Murugan et al., 2023; Stanski et al., 2023). Insights derived from studies on TIMP2 and IGFBP7, which modulate cell cycle, exhibit differential expression and distribution, and undergo alterations in severity of AKI, along with changes in protein distribution, are crucial for guiding the diagnosis of renal injury across various etiologies, extents, and locations (proximal tubule, distal tubule, collecting duct, or interstitium). In 2013, was identified by Kashani et al. (2013) as a biomarker for AKI(56). From a screening of 340 candidate biomarkers, IGFBP7 was found to predict AKI based on creatinine standards. Released from proximal tubules, IGFBP7 facilitates pinpointing specific segments of damaged renal tubules. In the early phases of cellular stress, IGFBP-7 and TIMP2 induce G1 cell cycle arrest by inhibiting cyclin-dependent protein kinases. A *TIMP2*×*IGFBP7* > 0.3 has demonstrated a sensitivity of 92% for moderate to severe AKI (Luthra and Tyagi, 2019; Zaouter and Ouattara, 2019). Moreover, elevated *IGFBP7* mRNA levels have been observed in uranium nitrate-induced acute renal failure in mice (Taulan et al., 2006).

The mechanism of IGFBP7 in AKI involves its ability to regulate cell cycle progression (Zang et al., 2019), inflammation, fibrosis, apoptosis, and oxidative stress (Yu et al., 2022). IGFBP7 induces G1 phase cell cycle arrest in renal tubular epithelial cells, thereby inhibiting their proliferation. This effect is mediated through the upregulation of CDK inhibitors such as p21 and p27, which suppress CDK activity, thus halting cell cycle progression (Wang et al., 2019). Additionally, IGFBP7 is implicated in the modulation of renal inflammation and fibrosis, hallmark features of AKI progression. It can regulate the expression of pro-inflammatory cytokines and chemokines, including IL-6 and TNF-α, thereby mitigating inflammatory responses in the kidney (Zwaag et al., 2019). Moreover, IGFBP7 has been shown to inhibit the activation of the TGF-β signaling pathway (van Duijl et al., 2022), a key mediator of renal fibrosis, thereby ameliorating fibrotic changes in the kidney. In summary, IGFBP7 serves as a promising biomarker for acute kidney injury, aiding in early detection and prognosis assessment. Its involvement in regulating cell cycle progression, inflammation, fibrosis, apoptosis, and oxidative stress underscores its significance in AKI pathogenesis and highlights its potential as a therapeutic target.

## 7 The role of IGFBP7 in reproduction

**FIGURE 3:**
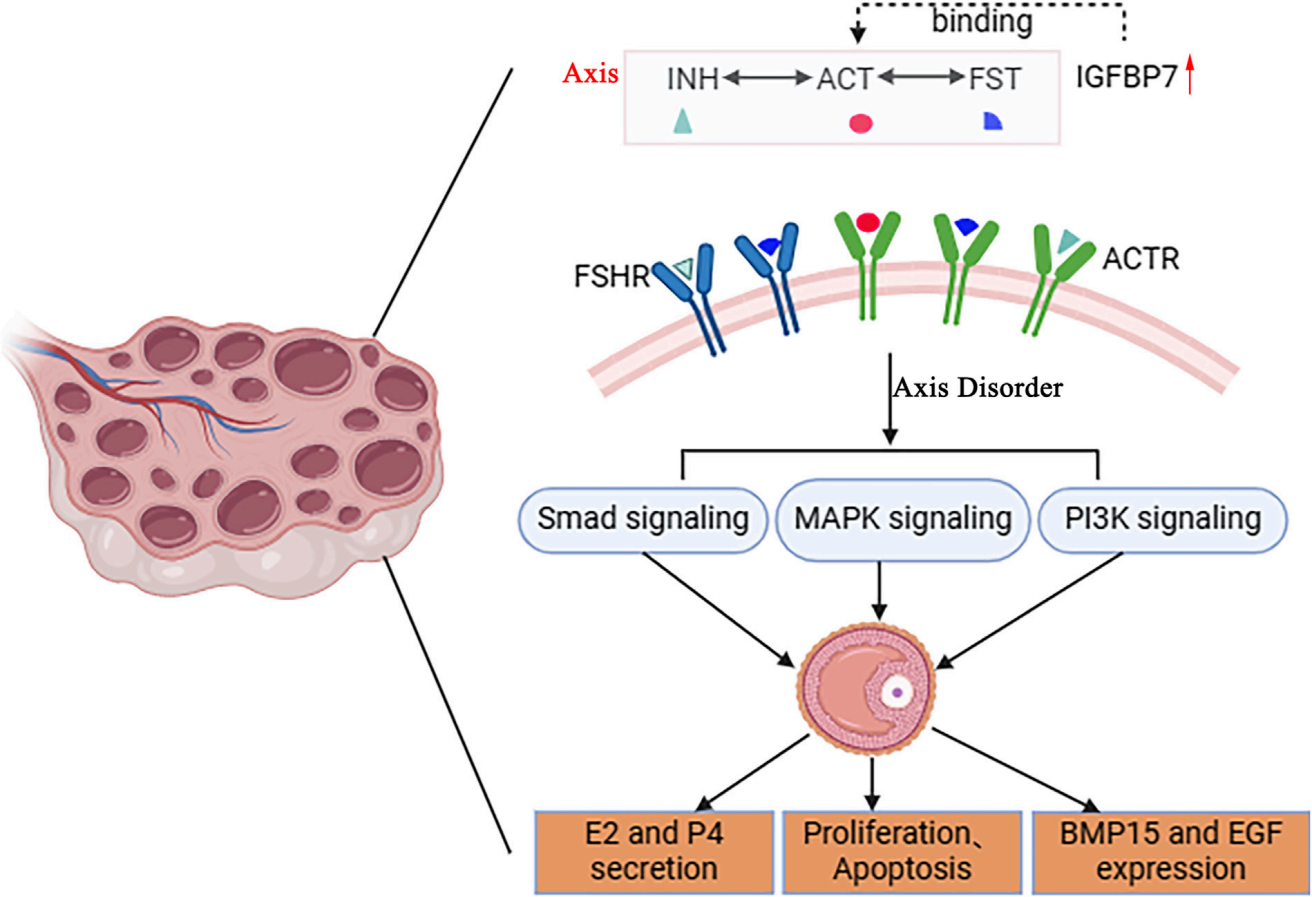
Potential mechanism of IGFBP7 regulation in follicular development. IGFBP7 is structurally homologous to follistatin (FST) and has been shown to interact with activins (ACT) on protein level. The INH-ACT-FST axis plays a crucial role in regulating follicular development, modulating follicular cell secretion of E2 and P4, proliferation, apoptosis, and expression of BMP15, EGF, through Smad, MAPK, and PI3K signaling pathways. This schematic diagram illustrates the potential molecular mechanism by which IGFBP7 regulates follicular development, highlighting its interactions with key players in the INH-ACT-FST axis and downstream signaling pathways.

IGFBP7 plays a regulatory role in folliculogenesis (Wijesena et al., 2024). *IGFBP7* exhibits significant homology with follicular inhibin (Kato, 2000). Follicular inhibin is considered an inhibitor of FSH secretion, playing a pivotal role in follicular development and ovarian function (Appiah Adu-Gyamfi et al., 2020). Similarly to follicular inhibin, IGFBP7 can bind with activin A, thereby influencing the growth inhibitory effects of the TGF-β superfamily on granulosa cells (Figure 3) (Tamura et al., 2007). Recent studies have shown that *IGFBP7* is expressed in granulosa cells of pig antral follicles and bovine corpora lutea, capable of suppressing estrogen production in granulosa cells (Ożegowska et al., 2018). RNA-seq results have revealed high expression of *IGFBP7* in granulosa cells of buffalo antral follicles, and expression of *IGFBP7* has been identified in granulosa cells of bovine large antral follicles and bovine corpora lutea (Li et al., 2018). Knockdown of *IGFBP7* has been observed to affect the number of apoptotic cells, cell cycle, cell proliferation, as well as estrogen and progesterone production (Kim et al., 2018). Treatment of granulosa cells with FSH and activin has significantly increased the expression of *Cyp19a1* mRNA and secretion of 17β-estradiol (E2), whereas the addition of exogenous recombinant mouse IGFBP7 in the culture medium inhibits this promotion (Tamura et al., 2007). Treatment of granulosa cells with *IGFBP7*-specific small interfering RNA (siRNA) reduces *IGFBP7* expression, enhancing FSH-stimulated E2 secretion into the culture medium. These results suggest that IGFBP7 inhibits estrogen production in granulosa cells, indicating that this protein secreted into the follicular fluid may serve as an ovarian intrinsic factor, negatively regulating granulosa cell differentiation (Yoshie et al., 2021). Furthermore, invertebrate insulin-like growth factor-binding proteins (ILPBPs) share structural homology with vertebrate IGFBP7, and ILPBPs have been shown to potentially function in ovarian development in the invertebrate red deep-sea crab (Huang et al., 2021).

IGFBP7 is also significantly associated with embryo implantation and the success rate of pregnancy. IGFBP7 is present in the uterine glandular epithelial cells and uterine stromal cells, with elevated expression during the mid-to-late secretory phase of the menstrual cycle in women (Domínguez et al., 2003). *In vitro* studies have demonstrated that IGFBP7 acts as a decidualization regulator in uterine stromal cells, potentially exerting its effects during the decidualization process of uterine stromal cells (Kutsukake et al., 2007; Yoshie et al., 2021). IGFBP7 participates in embryo implantation and uterine decidualization. Inhibition of *IGFBP7* significantly increases the TH1-type cytokine IFNγ and decreases the Th2-type cytokines IL-4 and IL-10, thereby inhibiting uterine decidualization and reducing uterine receptivity. This can significantly lower embryo implantation and pregnancy rates, leading to pregnancy failure in a mouse model (Liu et al., 2012). In human umbilical vein endothelial cells, IGFBP7 treatment inhibits exogenous VEGF-induced angiogenesis, proliferation, and phosphorylation of MEK and ERK (Tamura et al., 2009). Using the human endometrial epithelial cell line (EM1) to study the significance of IGFBP7 in endometrial glandular function, the results indicate that IGFBP7 regulates glandular cell morphological changes by interfering with normal PKA and MAPK signaling pathways associated with the transformation and/or differentiation of endometrial glands, which is crucial for the initiation of embryo implantation (Kutsukake et al., 2010).

IGFBP7 plays a crucial role in pathological pregnancies, including complete hydatidiform mole, pregnancy-related nausea and vomiting (hyperemesis gravidarum), and endometriosis. IHC analysis revealed that downregulation of *IGFBP7* may play a significant role in the progression of complete hydatidiform mole (Xiao et al., 2014). Common variants of *IGFBP7* are susceptibility loci for the diagnosis of pregnancy-related nausea and vomiting (Fejzo et al., 2019b), with serum levels of IGFBP7 significantly increased in hyperemesis gravidarum women at 12 weeks of pregnancy (Fejzo et al., 2019a). Moreover, the homologue of fruit fly *IGFBP7* has been shown to play a role in coordinating neurons between metabolic states and feeding behavior, potentially conveying food preferences and pregnancy intentions (Bader et al., 2013). IGFBP7 is associated with the pathophysiology of endometriosis, as serum IGFBP7 concentrations in patients with endometriosis are significantly higher than those in the control group (Kutsukake et al., 2008), and metformin can upregulate the expression of *IGFBP7* in both human and mouse models of endometriosis (Huang et al., 2022). IGFBP7 is also involved in male reproductive processes. A study conducted at the Federal University of São Paulo from May 2014 to April 2016 detected increased expression levels of IGFBP7 protein in the semen of patients with varicocele using Western blot analysis (Belardin et al., 2016).

In summary, IGFBP7 plays a multifaceted role in folliculogenesis, embryo implantation, pregnancy success, and pathological pregnancies, including conditions like complete hydatidiform mole, hyperemesis gravidarum, and endometriosis. Its involvement in regulating decidualization, angiogenesis, glandular function, and neuronal coordination underscores its significance in reproductive processes and highlights its potential as a diagnostic and therapeutic target in reproductive disorders.

In this review, we focus on the roles of IGFBP7 in tumor development, acute kidney injury, and reproduction due to their significant impact on clinical outcomes and the extensive research supporting IGFBP7’s involvement in these areas. These functions are critical in understanding IGFBP7’s diverse biological activities and its potential as a therapeutic target. Furthermore, IGFBP7’s interaction with key signaling pathways such as the AKT/ERK pathway, which are common to these conditions, underscores its multifaceted role in cellular processes. In the realm of future research, IGFBP7 holds promise across multiple fronts. In cancer, its tumor-suppressive properties in melanoma, breast, and colorectal cancers beckon exploration into underlying mechanisms and its potential as a biomarker for early detection and therapeutic target. Additionally, its role in fibrosis regulation in organs like the liver, lungs, and kidneys warrants investigation into fibrotic disease pathogenesis and therapeutic potential. In metabolic disorders such as diabetes and obesity, IGFBP7’s influence on metabolic processes hints at diagnostic and therapeutic applications. By delving into these avenues, IGFBP7 could emerge as a pivotal player in disease diagnosis, prognosis, and treatment strategies, offering hope for improved healthcare outcomes.
